# Bacterial microbiome of faecal samples of naked mole-rat collected from the toilet chamber

**DOI:** 10.1186/s13104-022-06000-8

**Published:** 2022-03-18

**Authors:** Kah-Ooi Chua, Iqra Fatima, Yin Yin Lau, Kar Wai Hong, Wai-Fong Yin, Andrei Mardaryev, Kok-Gan Chan, Chien-Yi Chang

**Affiliations:** 1 Centre for Research in Biotechnology for Agriculture, University of Malaya, 50603 Kuala Lumpur, Malaysia; 2 Centre for Skin Sciences, School of Chemistry and Biosciences, University of Bradford, Bradford, BD7 1DP UK; 3 Department of Biotechnology, Faculty of Applied Sciences, UCSI University Kuala Lumpur, 56000 Kuala Lumpur, Malaysia; 4 International Genome Centre, Jiangsu University, Zhenjiang, China; 5 Institute of Biological Sciences, Faculty of Science, University of Malaya, 50603 Kuala Lumpur, Malaysia; 6 Institute of Marine Sciences, Shantou University, Shantou, 515063 China; 7 School of Dental Sciences, Faculty of Medical Sciences, Newcastle University, Newcastle upon Tyne, NE2 4BW UK

**Keywords:** Naked mole-rat, *Heterocephalus glaber*, Bathyergida, Coprophagy, Microbiome, Rodent, QIIME2, UniFrac

## Abstract

**Objective:**

The naked mole rats (NMRs, *Heterocephalus glaber*) are subterranean rodents that belong to the family Bathyergidae. They gained the attention of the scientific community for their exceptionally long lifespan of up to 30 years and have become an animal model of biomedical research on neurodegenerative diseases, aging and cancer. NMRs dig and survive in a maze of underground tunnels and chambers and demarcate toilet chambers for defecation and urination. Due to their coprophagic behaviours, we believed that the toilet chamber might play a role in maintaining optimal health of the NMRs. A 16S rRNA gene amplicon sequencing was performed to characterize the bacterial microbiome of faecal samples collected from the toilet chamber of a laboratory NMR colony.

**Results:**

Four faecal samples were collected at different time points from the same toilet chamber of a laboratory NMR colony for analysis. The 16S rRNA gene amplicon sequencing revealed that bacterial phyla Firmicutes and Bacteroidetes were the dominant taxa in the bacterial microbiome of NMRs. The relative abundance of the bacterial taxa shifted substantially between time points, indicating a dynamic microbiome in the toilet chamber. The data provided an insight to the faecal microbiome of NMRs in the toilet chamber.

**Supplementary Information:**

The online version contains supplementary material available at 10.1186/s13104-022-06000-8.

## Introduction

Resistance to age-related conditions (cancer, neurodegeneration, cardiovascular disease) and extraordinary life span with sustained health are hallmark characteristics of the naked mole rat (NMR; *H. glaber*), the longest-living rodent with a maximum lifespan of over 30 years [1]. Besides, NMR is a eusocial mammal with a well-organised colonial structure that consists of overlapping generations (up to 300 individuals), a single breeding female (the queen) and 1–3 breeding males. Meanwhile, the non-breeding members are sexually monomorphic with similar looking external genitalia and anogenital distance [2]. Wild NMRs thrive in a maze of underground tunnels and chambers. They exhibit a unique behaviour of demarcating activity chambers for feeding and resting, and toilet chambers for defecation and urination [3]. Interestingly, NMRs exhibit coprophagic behaviours (both autocoprophagy and allocoprophagy), which may be important to maintain their health, nutrients and eusocial behaviours [4]. Similar behaviours have also been observed among NMRs in captivity [5, 6]. Due to its unique biological and genetic features, NMR is getting increasing interest for biomedical research as an animal model.

Previous studies on the faeces of wild NMRs [7] and caecum of laboratory-grown individuals [8] have revealed complex microbiome compositions in NMRs. Due to their coprophagic behaviour, their gut microbiome may also be influenced by the intake faeces, which are normally discharged in the toilet chamber that lack microbiome information. Thus, to gain insight to the role of toilet chamber in maintaining optimal health in the rodent species, a microbiome analysis on the faecal samples from the toilet chamber is necessary. In this study, we collected faecal samples from the toilet chamber of a laboratory NMR colony at four different time points for a bacterial microbiome analysis. We also performed a comparative faecal microbiome analysis with the wild NMRs from a previous study [7] to identify the unique microbial features associated with the toilet chamber of NMR colony.

## Main text

### Materials and methods

#### Specimen collection

A NMR colony was maintained in interconnected chambers in Biological Services Unit at the University of Bradford (establishment licences X99822F50), at 28 to 30 °C ambient temperature, 45–60% humidity, 12 h red light/dark cycle to mimic an underground tunnel environment [3]. The NMRs were fed with fruits and vegetables. The whole faecal specimen in the toilet chamber of the colony was collected during the routine clearance at four time points as samples A (4th of Oct 2019), B (25th of Oct 2019), C (29th of Oct 2019) and D (1st of Nov 2019), by Institute of Animal Technology qualified animal technicians. Collected faecal samples were immediately preserved in liquid nitrogen, transported to the laboratory and stored in − 80 °C.

#### 16S rRNA gene amplification and Illumina sequencing

The faecal specimens were homogenized and the total DNA of the faecal specimens (200 mg) was extracted using a Qiagen QIAamp Fast DNA Stool Mini kit. The 16S rRNA gene (V3–V4 region) was amplified as previously described [9] using the primer pair: forward 5′-TCGTCGGCAGCGTCAGATGTGTATAAGAGACAGCCTACGGGNGGCWGCAG-3′and reverse 5′-GTCTCGTGGGCTCGGAGATGTGTATAAGAGACAGGACTACHVGGGTATCTAATCC-3′ (Illumina overhang adapter sequences were underlined) [10]. The 25 μl PCR reaction consisted of 1 × KAPA HiFi HotStart ReadyMix (KAPA Biosystems), 1 μm of each primers and 12.5 ng of DNA template. The PCR was performed using the program 95 °C for 3 min, 25 cycles of 95 °C for 30 s, 55 °C for 30 s, 72 °C for 30 s, followed by 72 °C for 5 min. The PCR product was verified for correct size (~ 550 bp) on a Bioanalyzer using a DNA 1000 chip and purified with Ampure beads (Agencourt Bioscience). An index PCR was performed following the manufacturer’s protocol, which consisted of 5 µl of purified PCR product, 1 × KAPA HiFi HotStart ReadyMix, 5 µl of index 1 and index 2 primers in 50 µl reaction. The PCR products were purified and verified for correct size on a Bioanalyzer (~ 630 bp). The 16S rRNA gene libraries were quantified, normalized and pooled into a combined library. The pooled 16S rRNA gene library was denatured and spiked with a 5% PhiX, before being sequenced on an Illumina MiSeq (2 × 250 bp paired-end read).

#### Sequence processing and data analysis

The demultiplexed paired-end reads were imported into QIIME2 version 2020.11 installed in a conda environment for data analysis [11]. The reads were quality-filtered using the QIIME2 plugin DADA2 before assigning them into amplicon sequence variants (ASVs) [12]. A rarefaction analysis was performed in QIIME2. All the ASVs were aligned using MAFFT [13] and the highly gapped regions were masked before construction of phylogeny using the FastTree2 [14].

The raw data of 16S rRNA gene amplicon sequencing for wild NMRs [7] were obtained from the authors or the NCBI SRA database for a comparative analysis. As the DADA2 denoising process is only applicable to single sequencing run at a time (https://docs.qiime2.org/2018.2/tutorials/fmt/), the datasets were quality-filtered in separate operations before merging with the NMR data from this study. The beta diversity indices weighted and unweighted UniFrac [15] were calculated and the bacterial microbiome differences between rodent groups were visualized following a principal coordinate analysis (PCoA). The EzBioCloud database [16] was used for taxonomic assignment of ASVs [17].

## Results

### Data summary

A total of 380,152 quality-filtered sequences were obtained from the four faecal samples collected at different time points from the toilet chamber of the laboratory NMR colony (average 96,177 per sample) (Additional file 1: Table S1). Majority of the sequences did not pass the quality filtering as we observed an overall low quality score for the raw sequence data from the QIIME2 Interactive Quality Plot. Nonetheless, a total of 1266 ASVs were identified and a rarefaction analysis indicated adequate coverage in capturing the bacterial diversity associated with the faecal samples as the number of observed ASVs of all samples reached plateau at a sampling depth of about 20,000 sequences (Additional file 2: Figure S1). Overall, a high bacterial diversity was detected in the faecal specimens (average 537 ASVs per sample) (Additional file 3: Table S2). Calculation of Shannon’s, Simpson’s and Chao1 diversity indices also showed proximate bacterial diversity associated with the faecal samples collected at different time points (Additional file 3: Table S2).

### Bacterial microbiome of the faecal samples from the toilet chamber of laboratory NMR colony

**Fig. 1:**
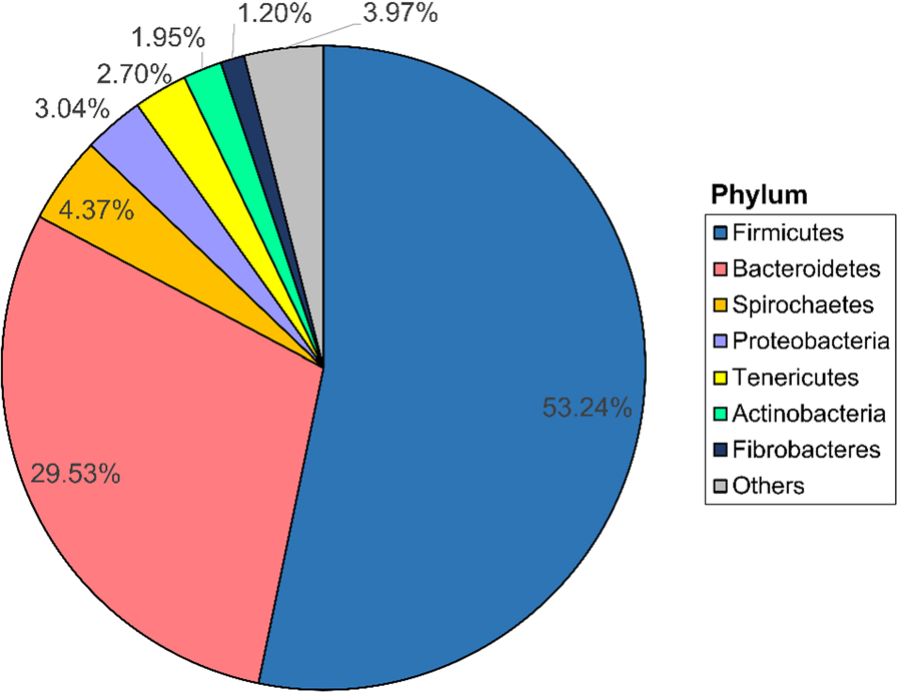
Pie chart showing the distribution of sequences into bacterial phyla, indicating the overall bacterial composition of the faecal samples collected from the toilet chamber of the laboratory NMR colony

With reference to the EzBioCloud database [16], majority of the sequences were assigned into two major bacterial phyla, Firmicutes (53.24%) and Bacteroidetes (29.53%) (Fig. 1). These were followed by bacterial phyla Spirochaetes, Proteobacteria, Tenericutes, Actinobacteria and Fibrobacteres at lower abundance (all at < 5%) (Fig. 1). The remaining ASVs were assigned into multiple bacterial phyla that all together represented only 3.97% of the total sequences (Fig. 1).

**Fig. 2:**
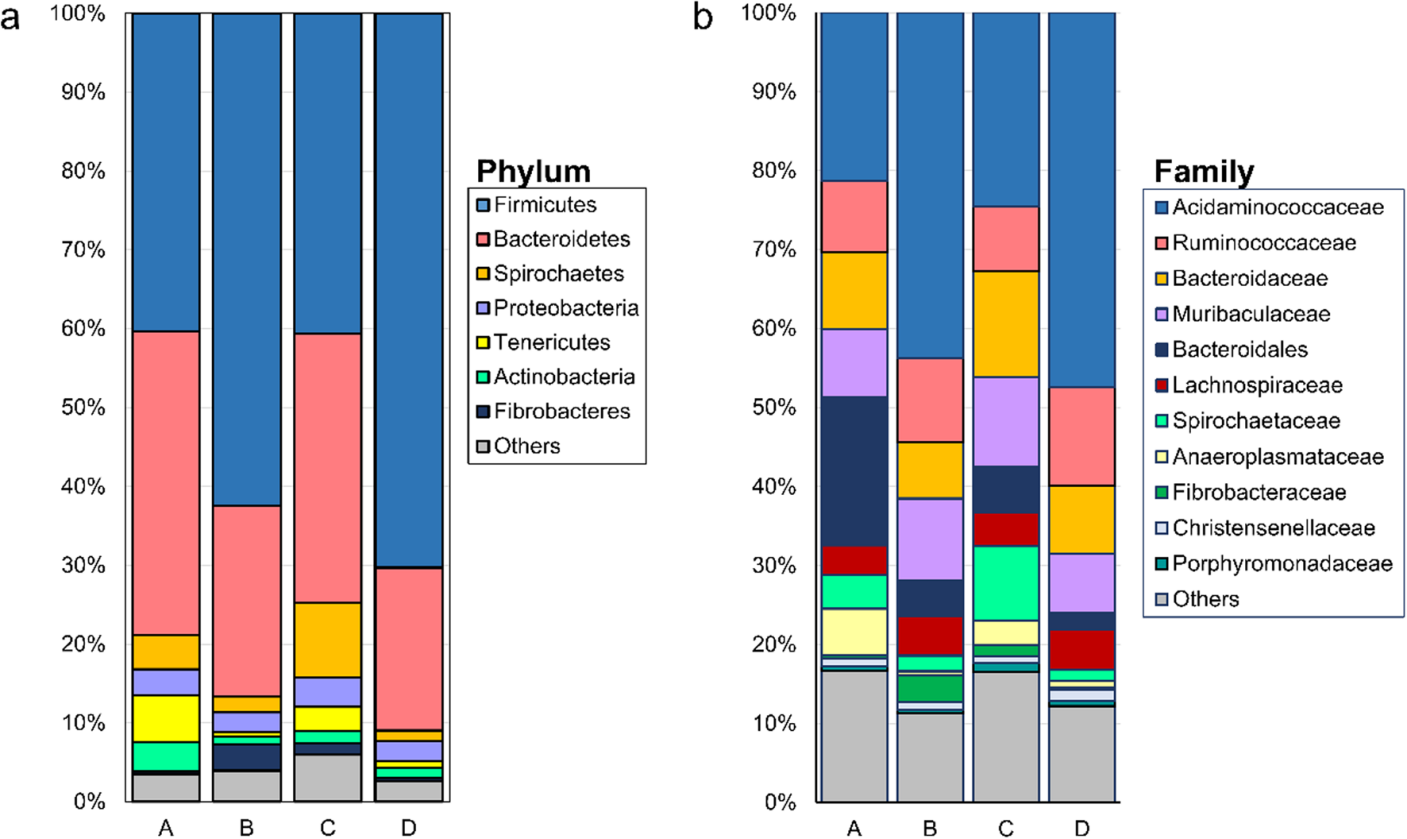
Relative abundance of bacterial taxa at the rank of phylum (**a**) and family (**b**) in the microbiome of faecal samples collected from the toilet chamber of laboratory NMRs

Similar bacterial taxa were consistently present in the NMR faecal samples collected at four different time points from the toilet chamber (Fig. 2a). Most of the variations between the samples were attributed to differential abundance of the bacterial taxa. For examples, at the phylum rank, a large difference was observed in the relative abundance of phylum Firmicutes between the faecal samples from different time points (40.37–70.29%) (Fig. 2a). Meanwhile, the remaining phyla showed fluctuated abundance but at a smaller margin among the four samples (Fig. 2a). Most of these phyla consisted of multiple ASVs assigned to different bacterial families. Acidaminococcaceae represented the most abundant bacterial family in the phylum Firmicutes and also the most abundant taxon in the faecal microbiome of toilet chamber in the NMR colony (Fig. 2b).

### Comparison of the faecal samples from the wild NMRs and the toilet chamber of laboratory NMR colony

The faecal microbiomes of wild NMRs (dataset from Debebe et al. [7]) were obtained for a comparative analysis with faecal microbiomes from the NMR’s toilet chamber in this study (Additional file 4: Table S3). Overall, all the NMR faecal microbiomes were dominated by bacterial phyla Firmicutes and Bacteroidetes (Additional file 5: Figure S2). The faecal microbiome differences between the NMR groups were more noticeable at the bacterial family level. The faecal microbiomes of the toilet chamber from laboratory NMRs was characterized by highly abundant family Acidaminococcaceae (21.26–47.35%) that was absent from the wild NMRs. Meanwhile, the wild NMRs possessed a high relative abundance of families Prevotellaceae, Lachnospiraceae and Spirochaetaceae in their microbiomes which was distinctive from the faecal samples of toilet chamber in the laboratory NMR colony (Additional file 6: Figure S3). The remaining bacterial families occurred at lower relative abundance and showed inconsistency in both the NMR groups, regardless of their living environments.

**Fig. 3:**
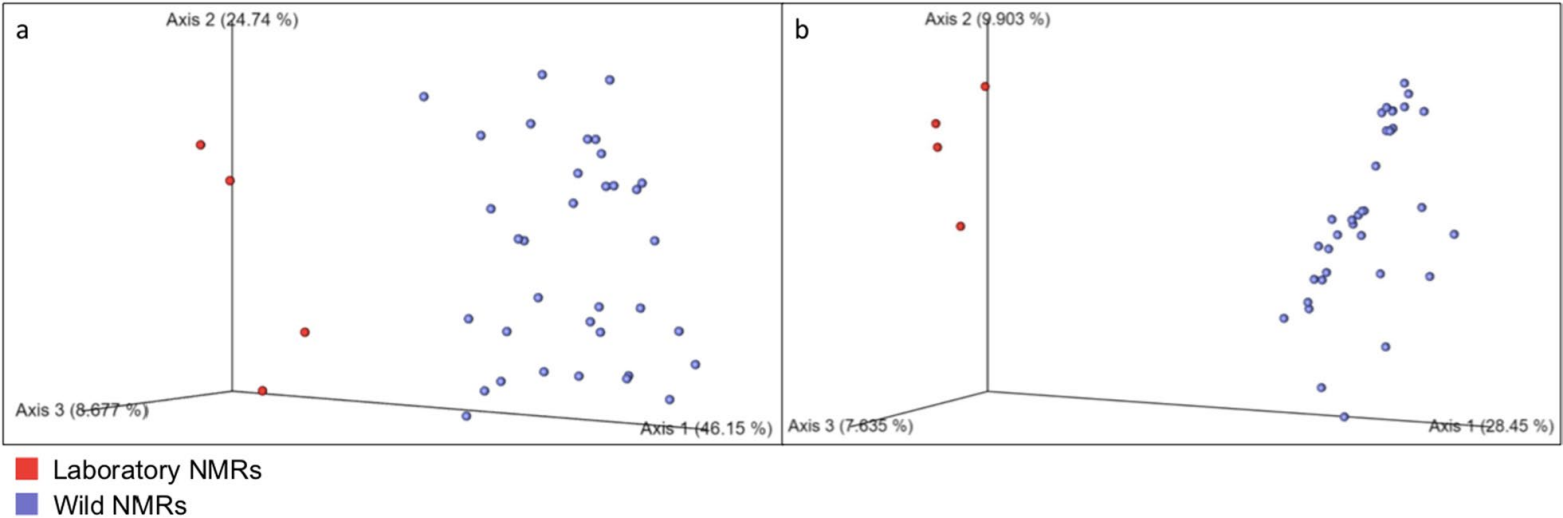
Principal coordinate analysis (PCoA) based on weighted (**a**) and unweighted (**b**) UniFrac distances of the bacterial microbiomes between the laboratory and the wild NMRs

The bacterial microbiome difference of the two groups of samples was then assessed using UniFrac analyses. From both weighted (Fig. 3a) and unweighted (Fig. 3b) UniFrac analyses, the faecal samples from the toilet chamber of laboratory NMR and the wild NMRs formed wide and heterogenous clusters of their own with significant group distance in the PCoA plots. These showed that the faecal samples of NMRs from different living environments (wild or laboratory) consisted of distinctive microbiomes.

## Discussion

The microbiomes of faecal samples from the NMR’s toilet chamber comprised of major bacterial phyla Firmicutes and Bacteroidetes (Fig. 2) that were also reported in the faecal microbiomes of wild NMRs [7] and cecal microbiome of laboratory NMRs [8]. Although all the faecal samples collected at different time points were detected with the same major bacterial taxa, the relative abundance of the taxa varied significantly between the samples. This revealed a dynamic microbiome in the toilet chamber with significant shifts in bacterial composition from time to time, despite a constant external laboratory environment (humidity, ambient temperature, light) with food supply in which the NMR colony was maintained.

The variation between time points in the microbiome of the toilet chamber could be influenced by the external environment. NMRs thrive in extensively interconnected underground tunnels and chambers which extend up to few kilometres long and 2 m underneath the surface [18]. They exhibit high hypoxia tolerance and capability in regulating their metabolic rate in the hypoxic underground nest environments [19]. When NMRs exhaust the available oxygen, it causes a prolonged period of oxygen deprivation in the underground nest, including the toilet chamber [18]. The fluctuation in oxygen level could alter the growth of bacteria in the faeces, leading to varying abundance levels of bacterial taxa in the faecal microbiome of the toilet chamber.

Besides, NMRs are strict herbivore that mainly forage underground for roots, bulbs, corms and tubers [20]. Their plant-based diets are generally cellulose-rich but low in proteins [5]. The retention of these food in their gut and cecum allowed active microbial fermentation and produced faeces with abundant microorganisms [21, 22]. Further microbial fermentation might happen when these faeces were excreted in the toilet chamber, creating a dynamic microbiome. Through coprophagy (both auto- and allocoprophagy), the faeces in the toilet chamber might confer nutritional advantage to NMRs by providing a protein source in the form of digested microbes [22].

In addition, we also acknowledged that excretion in the toilet chamber by the NMRs allowed the faeces to mix with the surrounding microorganisms. Through coprophagic practice, the NMRs could ingest these faeces that had been supplemented with environmental microorganisms in the toilet chamber. In another word, the NMRs could uptake new microorganisms from the environment that were beneficial for digestion and avoid homogeneity in their bacterial microbiome. Notably, preventing coprophagy in Brandt’s vole (*Lasiopodomys brandtii*) decreased the gut microbiota diversity of the rodent species [23].

On the other hand, we compared the faecal microbiomes of the toilet chamber to the wild NMRs [7] and revealed significantly different bacterial microbiomes between the two groups. The results indicated that captivity might constitute an influencing factor on the bacterial microbiomes of NMRs, which could relate to the changes in diet and environment in the laboratory. In fact, manipulation of diets is known to affect the gut microbiome of mice [24, 25], but similar study on NMRs is required to test the hypothesis.

Besides, the faecal microbiome differences between the toilet chamber in our study and the wild NMRs [7] could also be attributed to the use of different DNA extraction methods and sequencing settings. Debebe et al. [7] had used a bead-beating technique and a 2 × 300 bp paired-end setting in their study [7]. Although studies have shown that the impact of DNA extraction method on microbiome analysis was minor [26, 27], it was reported that a more accurate bacterial community structure was obtained using a bead-beating DNA extraction protocol [28]. The difference in DNA sequencing strategy (2 × 250 bp vs 2 × 300 bp) also limited deeper comparative analysis at the ASV level to determine the core microbiome of both the faecal samples, as the length differences would result in different ASVs from QIIME2 analysis.

## Conclusions

This study explored the bacterial microbiome of faecal samples collected from the toilet chamber of a laboratory NMR colony. The data revealed substantial shifts in terms of relative abundance of the bacterial taxa between the faecal samples collected at different time points, indicating a dynamic microbiome in the toilet chamber. In summary, this finding raised the possibility that the toilet chamber functions as a site for active microbial fermentation, which provide the NMRs with essential proteins in the form of digested microorganisms through their coprophagic behaviour. Overall, the dataset complemented other microbiome studies of NMRs in understanding the role of the toilet chamber and coprophagic behaviour in maintaining optimal health of the rodent species.

## Limitations

We acknowledged that the low number of faecal samples collected at different time points (only four) was inadequate in capturing a holistic microbiome variation in the toilet chamber of NMR colony. The lack of access to the toilet chamber of additional laboratory or wild NMR colony restricted further inter-colony analysis or microbiome comparison of the toilet chambers in the same study. Although we included a gut microbiome dataset from wild NMRs of a previous study for analysis, the DNA extraction method and sequencing strategy that were different from our study limited deeper analysis at the ASV levels and only preliminary results could be reported from the comparative analysis. Additional samples, including both biological and technical replicates are also required to generate a more robust dataset.

## Supplementary Information

**Additional file 1: Table S1. **16S rRNA gene sequences of bacteria in the microbiome of the faecal samples from the toilet chamber of the laboratory NMR colony.

**Additional file 2: Figure S1.** Rarefaction curves evaluating the richness of ASVs in the bacterial microbiome of faecal samples collected from the toilet chamber of the laboratory NMRs.

**Additional file 3: Table S2.** Alpha diversity analysis after rarefaction to 70,000 sequences per faecal sample from the toilet chamber of the laboratory NMR colony.

**Additional file 4: Table S3.** The number of 16S rRNA gene sequences in the bacterial microbiomes of faecal samples from wild individual NMRs and the toilet chamber of a laboratory NMR colony. The data for wild NMRs were obtained from previous studies (1. Debebe T, Biagi E, Soverini M, Holtze S, Hildebrandt TB, Birkemeyer C, et al. Unraveling the gut microbiome of the long-lived naked mole-rat. Sci Rep. 2017;7(1):1–9).

**Additional file 5: Figure S2.** The bacterial microbiomes of faecal samples from wild individual NMRs and the toilet chamber of a laboratory NMR colony. Barplot showing the relative abundance of bacterial taxa at the rank of phylum.

**Additional file 6: Figure S3.** The bacterial microbiomes of faecal samples from wild individual NMRs and the toilet chamber of a laboratory NMR colony. Barplot showing the relative abundance of bacterial taxa at the rank of family.

## Data Availability

The sequencing reads generated from this study from the faecal samples of naked mole-rat collected from the toilet chamber were deposited in the National Center for Biotechnology Information Sequence Read Archive (NCBI BioProject accession number PRJNA726749). The raw data of 16S rRNA gene amplicon sequencing for wild NMRs [7] were obtained from the authors.

## Abbreviations

NMR: Naked mole rat
ASV: Amplicon sequence variant
PCoA: Principal coordinate analysis
