# Supplementary material for: Bacterial microbiome of faecal samples of naked mole-rat collected from the toilet chamber
Source: BMC Res Notes. 2022 Mar 18;15:107. doi: 10.1186/s13104-022-06000-8 (PMC8932300; doi:10.1186/s13104-022-06000-8)
Supplement: Supplementary file 1 — Additional file 1: Table S1. 16S rRNA gene sequences of bacteria in the microbiome of the faecal samples from the toilet chamber of the laboratory NMR colony. [file 13104_2022_6000_MOESM1_ESM.docx]

| Faecal sample | Forward reads | Reverse reads | Number of sequences (Post QC) | Number of sequences (after chimera removal) |
| --- | --- | --- | --- | --- |
| A | 1932237 | 1932237 | 99031 | 98096 |
| B | 1549686 | 1549686 | 73555 | 72203 |
| C | 1742174 | 1742174 | 101643 | 100510 |
| D | 1506678 | 1506678 | 110482 | 109343 |

**Table S1** 16S rRNA gene sequences of bacteria in the microbiome of the faecal samples from the toilet chamber of the laboratory NMR colony.
