# Supplementary figures and images for: Bacterial microbiome of faecal samples of naked mole-rat collected from the toilet chamber
Source: BMC Res Notes. 2022 Mar 18;15:107. doi: 10.1186/s13104-022-06000-8 (PMC8932300; doi:10.1186/s13104-022-06000-8)

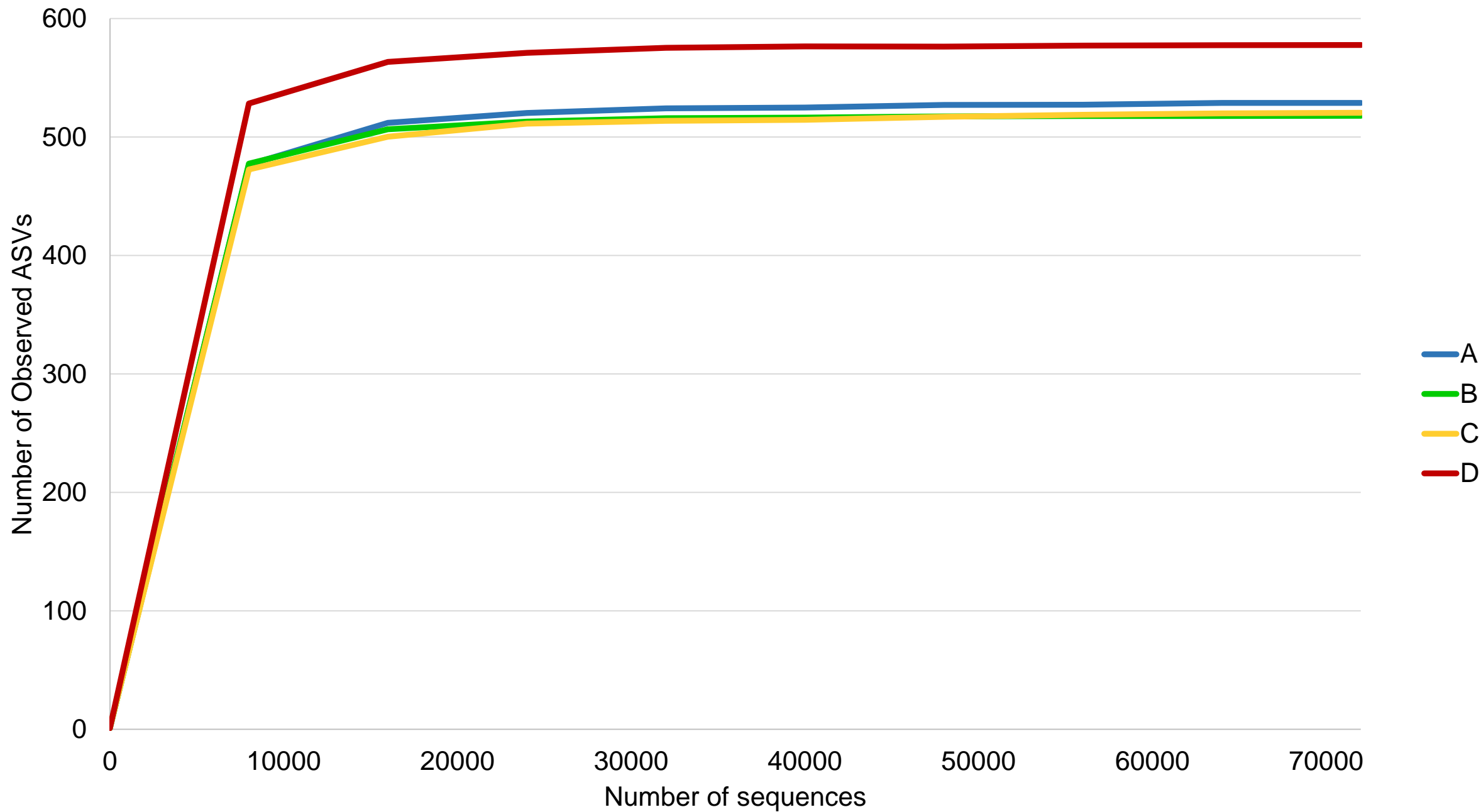

Supplement: Supplementary file 2 — Additional file 2: Figure S1. Rarefaction curves evaluating the richness of ASVs in the bacterial microbiome of faecal samples collected from the toilet chamber of the laboratory NMRs. [file 13104_2022_6000_MOESM2_ESM.pdf]

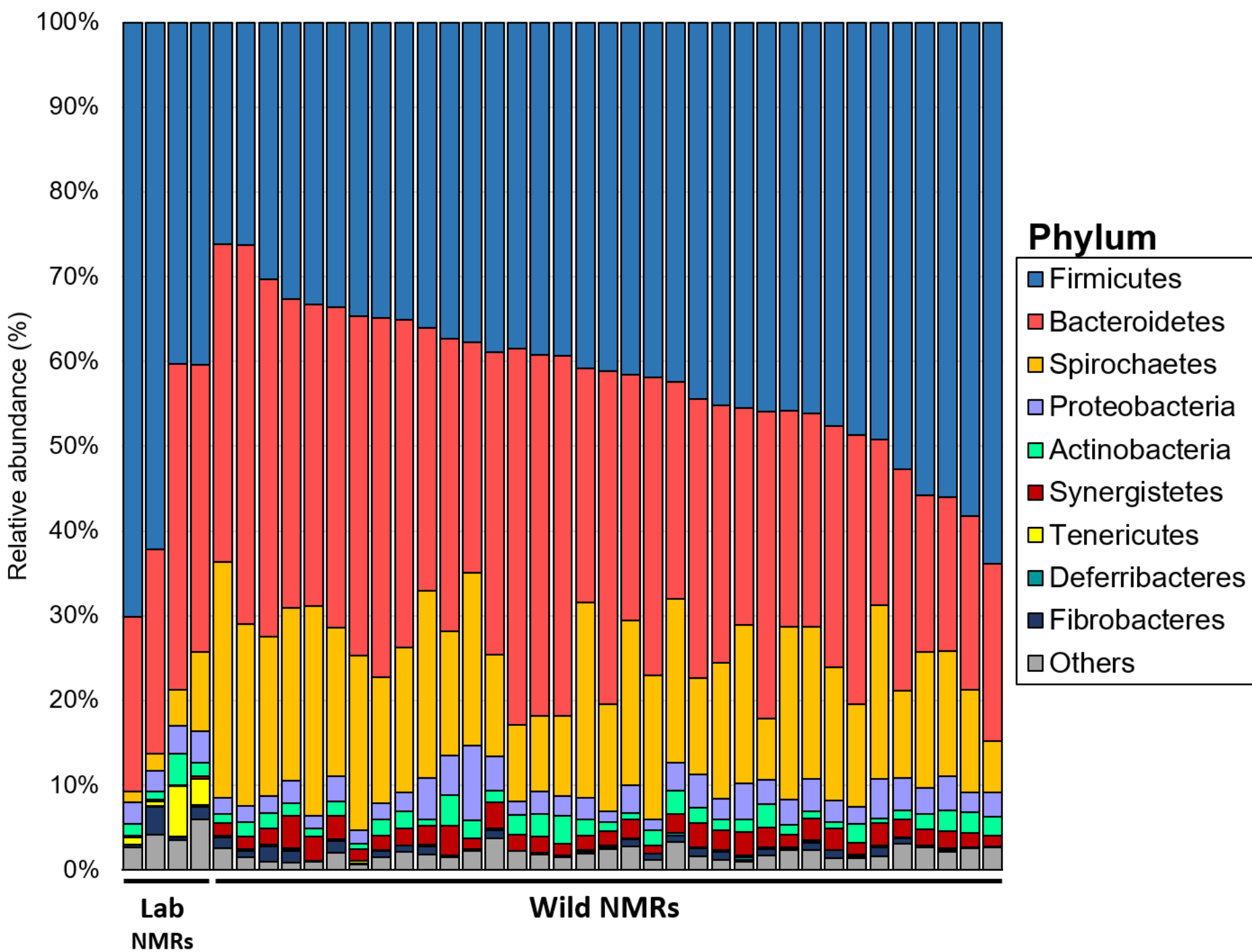

Supplement: Supplementary file 5 — Additional file 5: Figure S2. The bacterial microbiomes of faecal samples from wild individual NMRs and the toilet chamber of a laboratory NMR colony. Barplot showing the relative abundance of bacterial taxa at the rank of phylum. [file 13104_2022_6000_MOESM5_ESM.pdf]

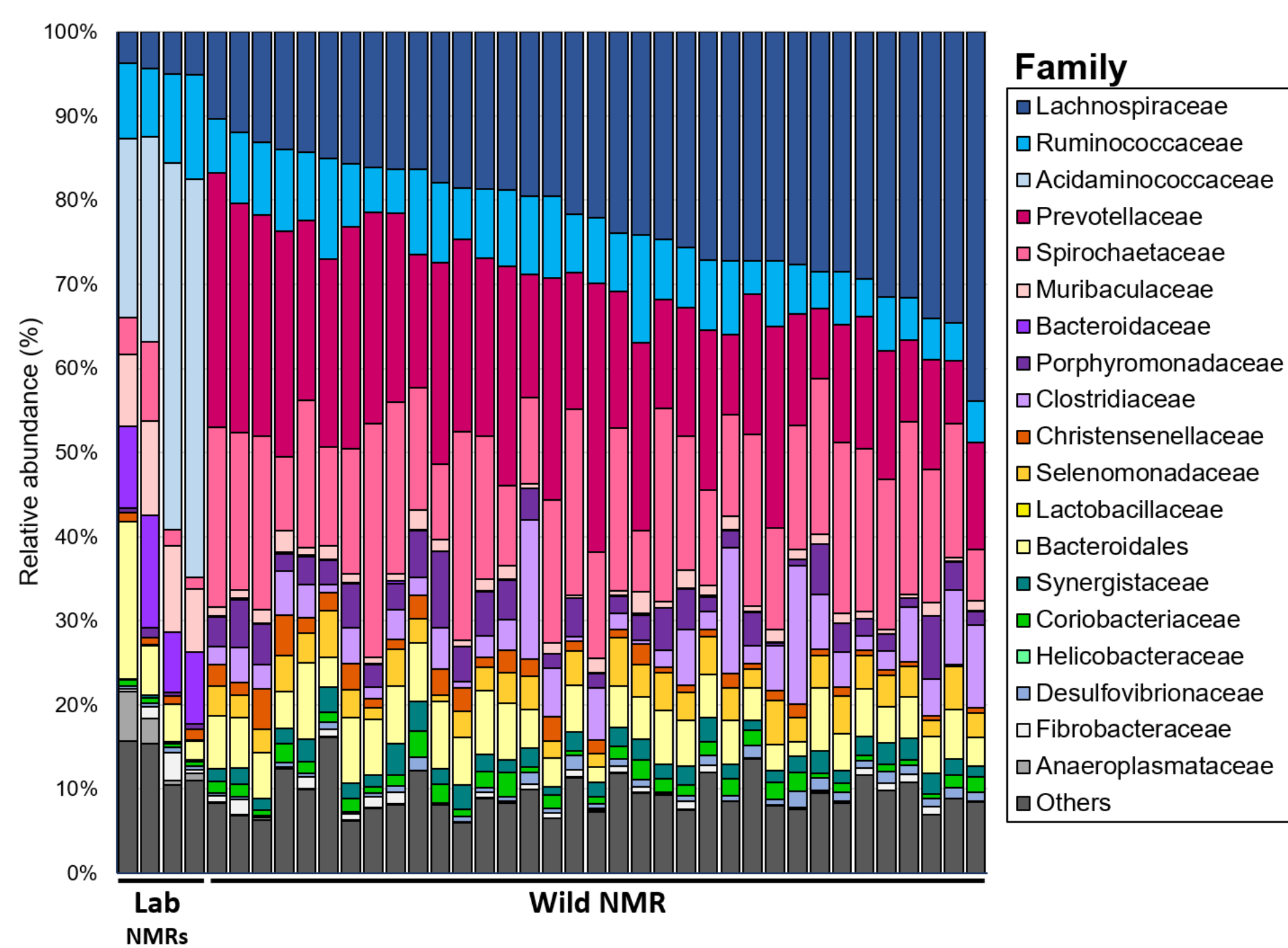

Supplement: Supplementary file 6 — Additional file 6: Figure S3. The bacterial microbiomes of faecal samples from wild individual NMRs and the toilet chamber of a laboratory NMR colony. Barplot showing the relative abundance of bacterial taxa at the rank of family. [file 13104_2022_6000_MOESM6_ESM.pdf]
