# Supplementary material for: Bacterial microbiome of faecal samples of naked mole-rat collected from the toilet chamber
Source: BMC Res Notes. 2022 Mar 18;15:107. doi: 10.1186/s13104-022-06000-8 (PMC8932300; doi:10.1186/s13104-022-06000-8)
Supplement: Supplementary file 3 — Additional file 3: Table S2. Alpha diversity analysis after rarefaction to 70,000 sequences per faecal sample from the toilet chamber of the laboratory NMR colony. [file 13104_2022_6000_MOESM3_ESM.docx]

| Faecal sample | Observed ASVs | Shannon | Simpson | Chao1 |
| --- | --- | --- | --- | --- |
| A | 530 | 6.87 | 0.98 | 544 |
| B | 518 | 6.62 | 0.97 | 518 |
| C | 523 | 7.17 | 0.99 | 539 |
| D | 577 | 6.73 | 0.97 | 578 |

**Table S2** Alpha diversity analysis after rarefaction to 70,000 sequences per faecal sample from the toilet chamber of the laboratory NMR colony.
