# Supplementary material for: Bacterial microbiome of faecal samples of naked mole-rat collected from the toilet chamber
Source: BMC Res Notes. 2022 Mar 18;15:107. doi: 10.1186/s13104-022-06000-8 (PMC8932300; doi:10.1186/s13104-022-06000-8)
Supplement: Supplementary file 4 — Additional file 4: Table S3. The number of 16S rRNA gene sequences in the bacterial microbiomes of faecal samples from wild individual NMRs and the toilet chamber of a laboratory NMR colony. The data for wild NMRs were obtained from previous studies (1. Debebe T, Biagi E, Soverini M, Holtze S, Hildebrandt TB, Birkemeyer C, et al. Unraveling the gut microbiome of the long-lived naked mole-rat. Sci Rep. 2017;7(1):1–9). [file 13104_2022_6000_MOESM4_ESM.docx]

| Laboratory/Wild NMRs | Sample ID | Faecal sample collection | Feature count |
| --- | --- | --- | --- |
| Laboratory | A | Toilet chamber | 98096 |
| Laboratory | B | Toilet chamber | 72163 |
| Laboratory | C | Toilet chamber | 100472 |
| Laboratory | D | Toilet chamber | 109343 |
| Wild | T1 | Individual NMR | 16650 |
| Wild | T10 | Individual NMR | 18288 |
| Wild | T11 | Individual NMR | 19709 |
| Wild | T12 | Individual NMR | 17353 |
| Wild | T13 | Individual NMR | 18212 |
| Wild | T14 | Individual NMR | 18071 |
| Wild | T15 | Individual NMR | 17899 |
| Wild | T16 | Individual NMR | 19011 |
| Wild | T17 | Individual NMR | 19577 |
| Wild | T18 | Individual NMR | 18295 |
| Wild | T19 | Individual NMR | 19376 |
| Wild | T2 | Individual NMR | 16465 |
| Wild | T20 | Individual NMR | 18197 |
| Wild | T21 | Individual NMR | 15080 |
| Wild | T22 | Individual NMR | 16141 |
| Wild | T23 | Individual NMR | 16420 |
| Wild | T24 | Individual NMR | 17566 |
| Wild | T25 | Individual NMR | 16904 |
| Wild | T26 | Individual NMR | 16767 |
| Wild | T27 | Individual NMR | 18649 |
| Wild | T28 | Individual NMR | 18656 |
| Wild | T29 | Individual NMR | 18743 |
| Wild | T3 | Individual NMR | 16496 |
| Wild | T30 | Individual NMR | 18847 |
| Wild | T31 | Individual NMR | 16824 |
| Wild | T32 | Individual NMR | 19281 |
| Wild | T33 | Individual NMR | 10255 |
| Wild | T34 | Individual NMR | 19603 |
| Wild | T35 | Individual NMR | 18373 |
| Wild | T4 | Individual NMR | 17170 |
| Wild | T5 | Individual NMR | 17421 |
| Wild | T6 | Individual NMR | 16912 |
| Wild | T7 | Individual NMR | 14818 |
| Wild | T8 | Individual NMR | 18844 |
| Wild | T9 | Individual NMR | 19244 |

**Table S3** The number of 16S rRNA gene sequences in the bacterial microbiomes of faecal samples from wild individual NMRs and the toilet chamber of a laboratory NMR colony. The data for wild NMRs [1] were obtained from the previous study.

1. Debebe T, Biagi E, Soverini M, Holtze S, Hildebrandt TB, Birkemeyer C, et al. Unraveling the gut microbiome of the long-lived naked mole-rat. Sci Rep. 2017;7(1):1-9.
